# The Spatial Shifts and Vulnerability Assessment of Ecological Niches under Climate Change Scenarios for *Betula luminifera*, a Fast-Growing Precious Tree in China

**DOI:** 10.3390/plants13111542

**Published:** 2024-06-02

**Authors:** Xian-Ge Hu, Jiahui Chen, Qiaoyun Chen, Ying Yang, Yiheng Lin, Zilun Jin, Luqiong Sha, Erpei Lin, El-Kassaby Yousry, Huahong Huang

**Affiliations:** 1 The State Key Laboratory of Subtropical Silviculture, Institute of Biotechnology, College of Forestry and Biotechnology, Zhejiang International Science and Technology Cooperation Base for Plant Germplasm Resources Conservation and Utilization, Zhejiang A&F University, Hangzhou 311300, China; xiangehu@zafu.edu.cn (X.-G.H.); chenn@stu.zafu.edu.cn (J.C.); chenqy6926@stu.zafu.edu.cn (Q.C.); yingy@stu.zafu.edu.cn (Y.Y.); yihenglin@stu.zafu.edu.cn (Y.L.); jin.zilun@foxmail.com (Z.J.); slq@stu.zafu.edu.cn (L.S.); zjulep@hotmail.com (E.L.); 2 Department of Forest and Conservation Sciences, Faculty of Forestry, The University of British Columbia, 2424 Main Mall, Vancouver, BC V6T 1Z4, Canada; y.el-kassaby@ubc.ca

**Keywords:** ensemble model strategy, habitat suitability, climate change, *Betula luminifera*

## Abstract

The spatial shifts and vulnerability assessments of ecological niches for trees will offer fresh perspectives for sustainable development and preservation of forests, particularly within the framework of rapid climate change. *Betula luminifera* is a fast-growing native timber plantation species in China, but the natural resources have been severely damaged. Here, a comprehensive habitat suitability model (including ten niche-based GIS modeling algorithms) was developed that integrates three types of environmental factors, namely, climatic, soil, and ultraviolet variables, to assess the species contemporary and future distribution of suitable habitats across China. Our results suggest that the habitats of *B. luminifera* generally occur in subtropical areas (about 1.52 × 10^6^ km^2^). However, the growth of *B. luminifera* is profoundly shaped by the nuances of its local environment, the most reasonable niche spaces are only 1.15 × 10^6^ km^2^ when limiting ecological factors (soil and ultraviolet) are considered, generally considered as the core production region. Furthermore, it is anticipated that species-suitable habitats will decrease by 10 and 8% with climate change in the 2050s and 2070s, respectively. Our study provided a clear understanding of species-suitable habitat distribution and identified the reasons why other niche spaces are unsuitable in the future, which can warn against artificial cultivation and conservation planning.

## 1. Introduction

Future agriculture and forestry will use integrated technologies such as remote sensing and geographic information system (GIS) to ensure that farms and plantations are more efficient, safe, and environmentally friendly. Indeed, the aggregate area of forest plantations in China has been on a consistent rise; nevertheless, the recent trajectory has exhibited a 19.75% decrement, dwindling from 10.1 × 10^4^ km^2^ (ninth national inventory) to 8.4 × 10^4^ km^2^ (eighth national inventory) [1,2,3]. Can we provide more reasonable protection and utilization strategies for perennial trees based on niche-based GIS modeling technology? At present, niche-based GIS modeling is commonly adopted to project species’ potential habitats, especially at large spatiotemporal scales [4,5,6]. Through niche-based GIS modeling theoretically, with the available species occurrence and environmental variables, which can calculate species niche characteristics in mathematical space consist of corresponding variables by using various mathematics algorithms [7,8]. It resulted in mapping the species’ potential distribution across landscapes and projecting its habitat distributions across space and time using GIS modeling technology.

Previously, several mathematics algorithms have been successfully used in niche-based GIS spatial simulations, each with their own advantages [9,10]. However, it should be stated that no single niche-based GIS modeling algorithm is appropriate for all future climate scenarios [11,12]. Hence, in order to decrease nondeterminacy due to various algorithms’ choice biases, an ensemble model (EM) strategy that integrates the message from individual niche-based GIS models and allocated various statistical algorithms has been offered. The EM strategy has already been proven to dramatically promote models’ precision and reliability [11,13,14,15]. Recently, it has been generally recommended that the species spread’s restrictions be examined in habitat estimation, particularly accounting for future variable environments during the modeling processes [16,17]. Generally, the environmental factors that restrict spread contain categorical variables (e.g., different types of soil). In the scope of the currently available niche-based GIS model technology, only maximal entropy algorithms (MaxEnt) could effectively handle categorical variables [18,19].

Precision forestry for sustainability and environmental protection cannot ignore the impact of rapid climate change. In fact, climate change has brought new uncertainties to the productivity of existing and future large-scale afforestation activities. The rapidly changing climate may move species’ suitable climate niches from their contemporary ranges, which is expected to cause maladapted and compromised plantation productivity [20] and broadly influence existing ecosystems and their socioeconomic activities [21,22]. Indeed, scientific evidence has proven that global warming is the chief reason for genetic diversity loss, suitable niche space fragmentation, and the changes in species niche spatial distribution [23,24]. Hence, predicting the changes in these tree plantation distribution areas will be of great practical and theoretical importance to studying the influence of climate change. On the other hand, given that environmental factors have perennially served as the primary driving force influencing the ecological niches of species, spatial shifts as well as ecological niche vulnerability assessments are necessary for plants, irrespective of the presence or absence of climate change.

*Betula luminifera* (H.) Winkl, Betulaceae, is a representative fast-growing and high-yielding valuable timber tree in China [25,26]. The species is well known for its high-quality timber, short juvenile phase, and fast growth. It is widely cultivated to provide commercial timber and promote the space quality of the environment based on ecological restoration and afforestation. However, the natural resources of *B. luminifera* are threatened by fragmentation from overharvesting and populations’ genetic diversity reduction [1,27,28,29]. Therefore, in 2015, to thoroughly maintain the sustainable development of forests, including *B. luminifera*, China prohibited commercial harvesting in natural forests, leading to a shortage in the domestic timber supply [1,30,31]. As a representative fast-growing timber plantation, *B. luminifera* has great development potential for addressing China’s future large timber gaps. However, rapid global warming has affected the climate variables in the original niche habitats of *B. luminifera* [17,24,32], which in turn are expected to increase the risk of maladaptation and productivity reduction.

Here, *B. luminifera*, a representative native plantation tree in China, was selected as the target to analyze habitat area (plantation) changes in contemporary and potential future climate scenarios using niche-based GIS modeling technology. For this objective, a comprehensive habitat suitability (CHS) model was developed to implement this analysis. During the procedure of model conformation, the EM strategy was performed to develop the climate suitability model and project the potential dynamic change under future climate scenarios. In addition, the maximal entropy algorithm was utilized to develop habitat limitation models to forecast spread restrictions. Our study is expected to provide a theoretical basis for *B. luminifera* planting and management, and the generated information is also expected to contribute to promoting domestic timber output.

## 2. Results

### 2.1. Model Performance and Key Environmental Variables

**Figure 1:**
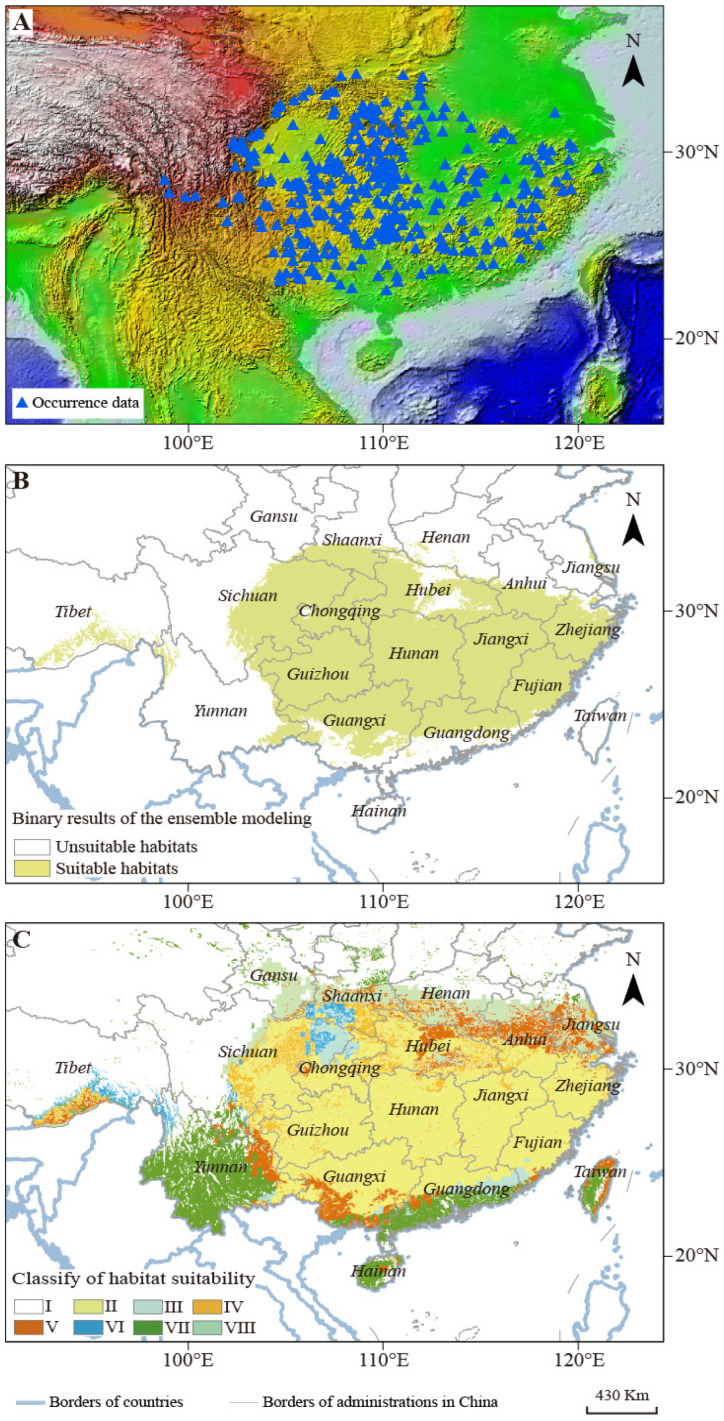
The geographical distribution of *B. luminifera* suitable habitats under the current climate condition. (**A**) the distribution of *B. luminifera* occurrence sample points; (**B**) the suitable habitats of *B. luminifera* developed by combining multi-climate model results. (**C**) The geographical distribution of different habitat-suitability types of *B. luminifera*.

To guarantee the reliability and validity of the occurrence data of *B. luminifera*, three standardized processing steps (described in material and method) were implemented. Finally, a total of 204 samples of *B. luminifera* existence information (with clear longitude and latitude) were retained to create the comprehensive habitat suitability model (Figure 1A and Table S1). 

**Figure 2:**
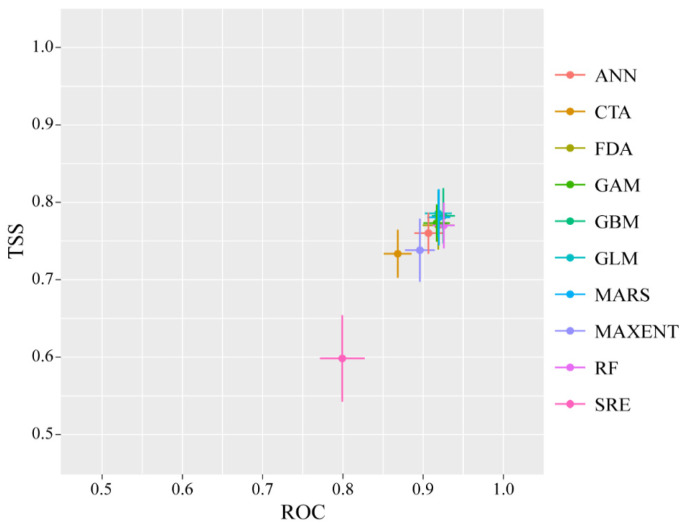
Mean model estimation values based on two different estimation metrics: the receiver operating characteristic (ROC) and the true skill statistic (TSS). SRE: surface range envelope, FDA: flexible discriminant analysis, GLM: generalized linear model, GAM: generalized additive model, MARS: multiple adaptive regression splines, GBM: generalized boosting model, CTA: classification tree analysis, ANN: artificial neural network, RF: random forest, and MaxEnt: maximum entropy.

Judging by the model’s evaluation results, all models with TSS values equal to or exceeding 0.60 and AUC values surpassing 0.85 deemed satisfactory (Figure 2). For each individual model, all of the ten simulation technologies were closely associated with the stable and efficient operation of each algorithm. And each model produced higher estimation values: the average TSS and AUC results were 0.749 and 0.900, respectively. Particularly, the MARS models exhibited the highest accuracy among the four algorithms, followed by GLM, GBM, and GAM (Figure 2). Hence, along with the satisfying TSS and AUC values, this shows that our ensemble model performs well and could offer accurate results. In addition, the soil and ultraviolet distribution limitation models also produced high AUC values, with 0.873 and 0.903, respectively. Therefore, the results of the climate suitability model and the distribution limitation model could be regarded as reasonable for *B. luminifera*.

**Figure 3:**
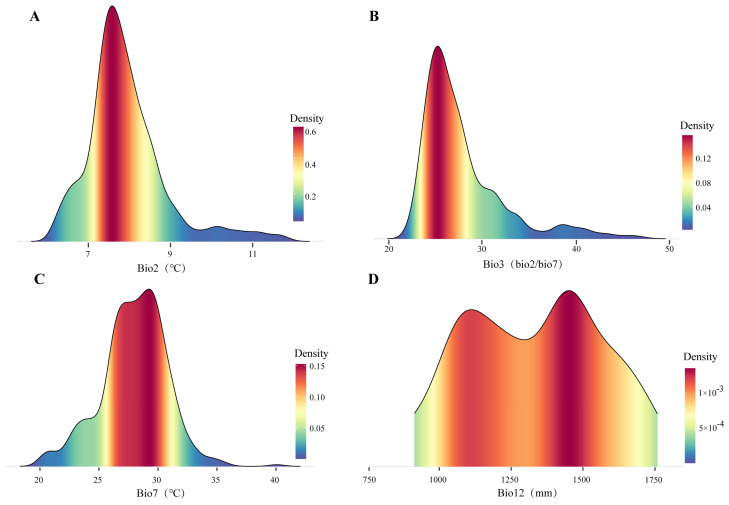
Kernel density plots for climatically related predictive variables in *B. luminifera* suitable habitats. (**A**) Mean diurnal temperature range (Bio2); (**B**) isothermality (Bio3); (**C**) temperature annual range (Bio7); (**D**) annual precipitation (Bio12).

Among the selected predictive bioclimatic variables (Table S2), eight climate factors related to temperature and precipitation were used to build the bioclimate suitability model of *B. luminifera* (Figures S1 and S2). The analysis of the climate factors’ contribution to model formulation reveals that Bio2, Bio3, Bio7, and Bio12 were the dominant bioclimatic variables (with a contribution rate of more than 10%) affecting the distribution range of the suitable habitat of *B. luminifera.* Then, the nonparametric Kruskal–Wallis multiple-range test was used to disclose the distinct adaptability ranges for these four dominant bioclimatic variables (Figure 3). Therefore, based on the frequency presentation pattern, we obtained the thresholds for the main bioclimatic parameters: the mean diurnal temperature range (Bio2) was from 7 to 8 °C; the isothermality (Bio3) range was from 24 to 28%; the temperature annual range (Bio7) was from 26 to 31 °C; and the annual precipitation range was from 1100 to 1600 mm (Figure 3).

### 2.2. The Geographical Scope of B. luminifera Suitable Habitat

The dynamic analysis showed that the core area (about 1.51 × 10^6^ km) of *B. luminifera* suitable habitat (Table 1) is mainly distributed in the south and southeast regions of China (Figure 1B). The distribution areas mainly covered Guizhou, Chongqing, northeast of Yunnan, southeast of Sichuan, and Tibet provenances (Yunnan–Guizhou Plateau area and Hengduan mountains); south of Shaanxi and Henan provenances (belongs to the southern slope of the Qinling Mountain); Hubei, Hunan, and Jiangxi, south of Anhui and Jiangsu provinces (Yangtze valley area); Fujian and Zhejiang provinces (Wuyi mountains area); and north of Guangxi and Guangdong provinces (belongs to the Nanling Mountains) (Figure 1B).

**Figure 4:**
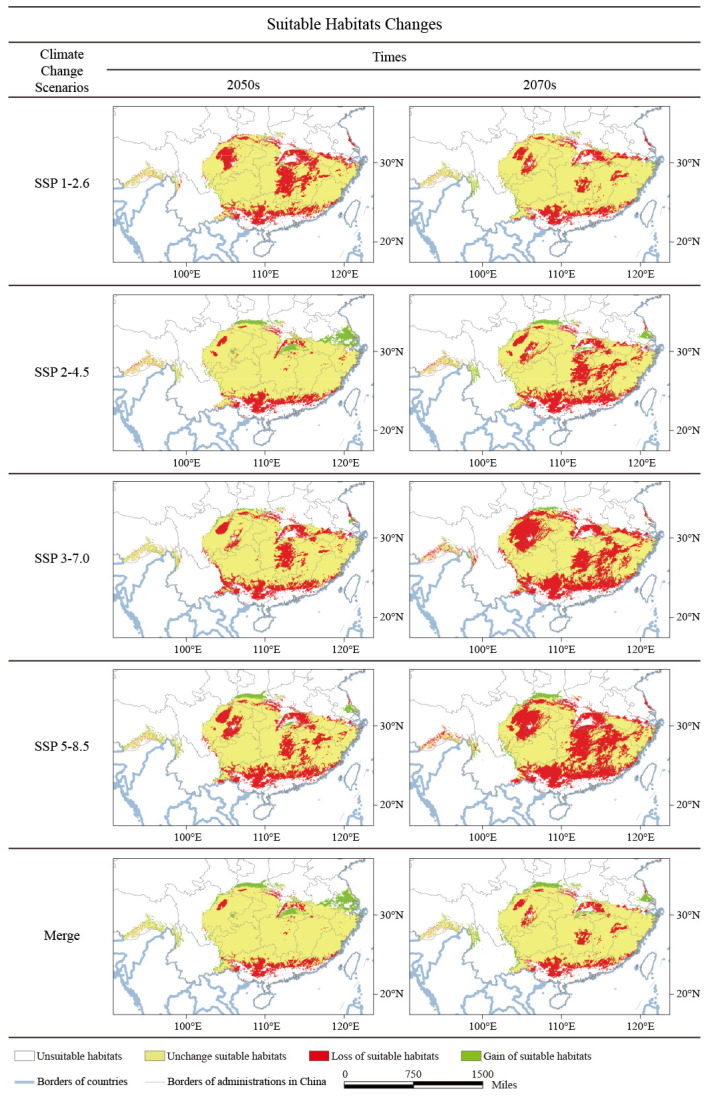
Projection of climatic habitat suitability for *B. luminifera* at various periods in China. Merge represents the intersection region of all possible suitable habitats at the same period.

By the 2050s, suitable *B. luminifera* habitats will shift towards the north of the current niche (Figure 4). Moreover, new potential distribution areas will appear in the south of Shaanxi, Henan, Anhui, Jiangsu, and northern Yunan province; the newly expended habitats are about 0.15 × 10^6^ km^2^ (Table 1). However, part of the current habitats is expected to be lost (about 0.18 × 10^6^ km^2^), mainly located in the south of Guangxi and Guangdong provinces, which are covered in the south of current habitats. Obviously, under these scenarios/periods, habitat areas with increasing and decreasing trends are almost equal, but the geography of the suitable habitats will shift towards higher altitudes.

With the extension of time, the trend of migration to higher latitudes of suitable habitat will continue in the 2070s, but the amplitude will decrease. New *B. luminifera* potential distribution areas will also appear in the north of the current niche, about 0.10 × 10^6^ km^2^ (Figure 4 and Table 1). However, it is worth noting that under these scenarios/periods, a large area of suitable habitats will be lost (0.25 × 10^6^ km^2^). Additionally, the degraded potential distribution will not only cover the south of the current habitats under baseline conditions, but it will also appear in the north of the current niche (Figure 4), which highlights the uncertainty brought about by climate change. Because the habitat areas with a decreasing trend are far beyond those with an increasing trend in the 2070s, the whole potential distribution area will considerably contract. Here, the changes in the area in potential habitat distribution under all climate change scenarios/periods are mapped (Figure 4 and Table 2), which clearly show the suitable habitat characteristics and dynamic migration trend of *B. luminifera* under future climate change scenarios.

### 2.3. The Spatial Distribution of Different Types of B. luminifera Habitats

According to the ecological niche model, along with comprehensive consideration of the limitations on soil and UV-B environment variables, a comprehensive habitat suitability (CHS) model was developed to estimate the *B. luminifera* suitable habitat distribution range, and this model provided the CHS value for every estimation grid. Hence, eight various habitat-suitability types of *B. luminifera* habitats were obtained in this study (Table 3).

In this study, the CHS results showed that *B. luminifera’s* suitable niche space in China will decrease if restrictive ecological variables are considered. Based on the area statistics results, the area of habitat with suitable climate, soil, and UVB conditions (II type) is about 1.15 × 10^6^ km^2^; the regions with suitable climate and soil environments but unsuitable UVB conditions (III type) are about 0.08 × 10^6^ km^2^; and regions including suitable climate and UVB environments, unsuitable soil conditions (IV type) are about 0.25 × 10^6^ km^2^ (Table 4). The optimal habitats (II type) were mainly located in Guizhou and Hunan provinces (Yunnan–Guizhou Plateau), Fujian and Zhejiang provinces (Wuyi mountains area) (Figure 1C). The most significant and highly suitable regions appeared in the Yunnan–Guizhou Plateau area and Wuyi mountains area, generally considered to be core *B. luminifera* production region.

**Figure 5:**
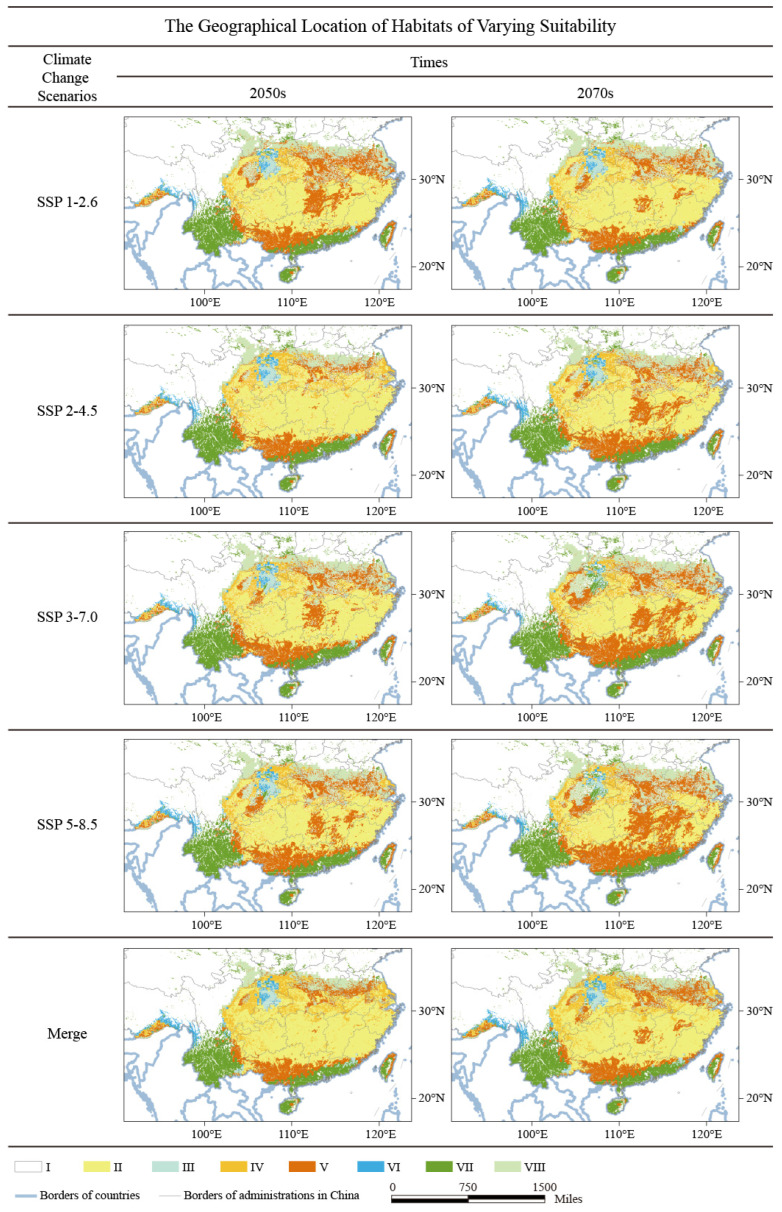
The geographical location of different types of suitable habitats for *B. luminifera* at various future climate change scenarios in China. Merge showing the intersection region of the suitable habitats at different scenarios. I: unsuitable; II: suitable habitat; III: habitats with an unsuitable UV-B condition; IV: habitats with an unsuitable soil condition; V: habitats with unsuitable climate conditions; VI: habitats with unsuitable soil and UV-B conditions; VII: habitats with unsuitable climate and UV-B conditions; VIII: habitats with unsuitable climate and soil conditions.

In the 2050s, the model forecast indicates that *B. luminifera’s* suitable habitats will shift towards the north and will slightly decrease (Figure 5). Large losses and degraded niche space will mainly appear in the southern of Nanling mountains regions (in the south of the current niche), containing Guangxi and Guangdong, and south of the Tibet provinces. In addition, some new potential distribution areas will appear in the north and northeast regions of China, mainly in northern Zhejiang and Hubei, Anhui, and Jiangxi provinces. The core production area of *B. luminifera* (suitable habitat type of II) will contract to 1.07 × 10^6^ km^2^ (a decreased of 6.95%).

In the 2070s, *B. luminifera*-suitable habitat areas will continue to move towards the higher latitude and decrease (Figure 5), since the loss of suitable niche space also occurs in the south of current areas in Guangxi and Guangdong provinces, but the location shifts further north than that of the 2050s. In addition, new suitable niche spaces will mainly appear in the south of Hubei, Anhui, and Jiangsu provinces. The core suitable habitat area of *B. luminifera* (suitable habitat type of II) will contract to 0.99 × 10^6^ km^2^ (a decrease of 13.91%). It is noteworthy that climate change will continuously impact *B. luminifera’s* suitable habitat distribution, leading to severe habitat fragmentation with the progression of time. Here, the characteristics and dynamic migration trends of these eight habitat-suitability types of *B. luminifera* are mapped in Figure 5, which clearly shows the changes in the area for each type under future climate change scenarios in China.

### 2.4. Changes in Potential Distribution Areas under Climate Change

**Figure 6:**
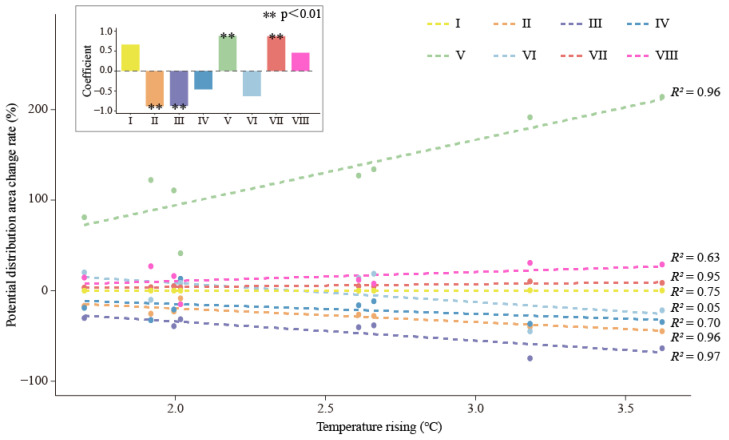
Area of habitats of differing suitability for *B. luminifera* at various periods in China. I: unsuitable; II: suitable habitat; III: habitats with an unsuitable UV-B condition; IV: habitats with unsuitable soil conditions; V: habitats with unsuitable climate conditions; VI: habitats with unsuitable soil and UV-B conditions; VII: habitats with unsuitable climate and UV-B conditions; VIII: habitats with unsuitable climate and soil conditions.

To quantify the influence of global climate change on the geographical range of these eight habitat-suitability types, the variance in annual average temperature was counted under various climate scenarios and stages. According to the above data, variations in the suitable habitat areas under various climate change scenarios were calculated (Table 5 and Table S3), and the dynamic forecast of the changing trends of the area variation for each suitable type was mapped (Figure 6). 

The results indicated that, with various predicted temperature increments, the area variation trend of these eight habitat-suitability types can be divided into three groups: with projected temperature increases, the potential distribution area shows a significant decreasing trend, including II, III, IV and VI (class one); a relatively steady trend, including I, VII, and VIII (class two); and an increase significantly trend, including V (class three) (Figure 6). Notably, the habitat-suitability type area changes in suitable habitat (II), habitats with unsuitable UV-B conditions (III), habitats with unsuitable climate conditions (V), and habitats with unsuitable soil and UV-B conditions (VII) were significantly associated with the different projected temperature increases.

## 3. Discussion

Future agrarian and silvicultural practices will harness integrated technologies (e.g., GIS) to assure sustainable conservation, heightened efficiency, and environmental congeniality on farms and plantations. Here, we have provided a more accurate assessment for the ecological niche of *B. luminifera* after considering limiting factors (soil and UV-B). The actual suitable niche constituted a mere 75.65% in comparison to scenarios where such factors were not considered. Furthermore, the spatial niche of *B. luminifera* is expected to be reduced with climate change, leading to a decrease in the entire spatial niche, suggesting the risk of maladaptation. Thus, forest management practices, such as in situ and ex situ protection/conservation and assisted migration, have the potential to curtail the expected niche losses and the maintenance of forest health and productivity, thereby sustaining future ecosystem services.

### 3.1. Ecological Characteristics of B. luminifera Habitats

A significant climatic characteristic in *B. luminifera* habitats is that the principal climate factors may be temperature-related (Figure 3). In order to clarify how global warming influences *B. luminifera’s* suitable habitat geographical range, the average value and standard deviation of the eight climate factors contained in the EM for various climate change scenarios and stages were counted (Table S4). The results indicated that, for the entire suitable habitats, the mean value of Bio1 was about 15.39 °C in current. In the next time period, the mean annual temperature would represent an upward trend in future climate scenarios, but the variation was different. In the 2050s, the annual mean air temperature (Bio1) value in the entire suitable habitats may equal 17.53 °C, and by the 2070s, Bio1 may reach 18.18 °C, meaning that the temperature increment will be 3.37 °C. In addition, the annual precipitation (Bio12) exhibited a similar upward trajectory, albeit with a markedly smoother progression: in the 2050s, the average Bio12 could retain a relatively steady trend; in the 2070s, the added precipitation will reach 88.41 mm. Bio2, Bio3, Bio4, Bio8, Bio15, and Bio18 have a high probability of showing a steady or slight increase, indicating that the seasonal tendencies of temperature and rainfall may remain essentially steady in the species’ entire suitable habitats.

Our analysis showed that the change in *B. luminifera’s* suitable habitat area was closely related to the projected temperature increases, which is consistent with other fast-growing tree plantation species affected by climate change. For example, there are at least 12 fast-growing timber tree species with potential distribution areas showing dynamic changes with different projected temperature increases, e.g., *Betula platyphylla* (a close relative species with *B. luminifera*) and *Cunninghamia lanceolata* (an important plantation timber tree in China) [6]. In addition, under the maximum temperature rise’s scenario (e.g., SSP585), most of the fast-growing tree plantation species will be shifting their suitable niche space to the north, and a large area of suitable habitat loss, especially in the south of the current niche under the current climate scenario, will cause severe habitat fragmentation [6]. Such phenomena align with our research, and the findings in this study highlight the variations in habitat areas for a representative fast-growing and high-yielding unique valuable timber plantation under the impact of predicted future climate warming, which will provide more information for reasonable afforestation under climate change in the future.

### 3.2. Limitative Effect of Soil and UV-B on B. luminifera Suitable Habitats

*B. luminifera* is a perennial woody plant. Unfit soil and ultraviolet types will restrict the extension of plantations. In this study, soil factors were found to be the major variables that were restricting the range of *B. luminifera*, particularly in Jiangsu, Anhui, Hubei, and the east of Sichuan provinces (the suitability type IV). Along with the suitable climatic environments, the range of unsuitable soil conditions (IV) was 0.25 × 10^6^ km^2^, which is 44% of the core *B. luminifera* habitats. Soil has already been proven to be an important factor in limiting plant growth and influencing ecosystem productivity [33,34]. Furthermore, an in-depth understanding of the intricate interplay between soil and climate on plant characteristics reveals that the primary variations are reflected in size disparities at both organ and plant levels, as well as in leaf development, delicately balancing leaf longevity with plant growth potential [35]. On the other hand, ultraviolet B radiation, a significant macroclimatic element exhibiting substantial variations across extensive spatial expanses [36], has been empirically shown to exert considerable influence on plant germination and growth [37,38]. Recently, the on-going changes in climate are increasingly exposing plants to novel combinations of UV-B and other climate factors, e.g., water availability and temperature [39,40]. In this study, the range of unsuitable UV-B conditions (III) was 0.08 × 10^6^ km^2^ (7% of the core *B. luminifera* production region), which is mainly located in southwest China (Figure 5), demonstrating that the dominant variables restricting the expansion of the species’ suitable niche space in these regions are likely to contain UV-B. However, it is not yet clear how soil and UV-B specifically regulate plant growth, and the physiological mechanism also needs further investigation.

The scope of environmental factors is likely to change due to the impact of future climate change. In this study, the decrease in suitability type IV indicates that the most suitable habitat of *B. luminifera* is shrinking under the influence of climate change. In addition, the suitability types III and IV also indicate that the newly added suitable environment is caused by climate change, maybe mostly located in unsuitable soil or ultraviolet conditions. In this study, the characteristics and dynamic migration trend of eight habitat-suitability types for *B. luminifera* were mapped (Figure 5), which clearly shows the geographic location of the restriction factor for each type under future climate change scenarios in China. Therefore, forest managers can formulate detailed vegetation protection and utilization strategies according to the changes in suitable habitat types in specific regions. For example, in central China, the range of habitat type IV (soil as limiting factors) will gradually decrease and significantly shift northward, and the new regions that have suitable niche space will appear to the north of Hubei and Anhui, in Chongqing provinces.

### 3.3. Management Priorities of B. luminifera under Climate Change

To prevent genetic diversity loss and ecological degradation in *B. luminifera* habitats, the reasonable designation of planting regions will be a key bond based on the species survival rate with respect to climate warming. Under natural conditions, population gene flow, migration, and vegetation succession are complicated processes that typically happen over a long period of time. It is generally accepted that the natural shifting of forest trees lags far behind the current rapid rate of climate change [5,41,42]. In a previous study, a significant linear positive correlation between the afforestation survival rate and the potential probability of presence by projecting the habitats distribution based on ecological niche models was found [43]. Concerning *B. luminifera*, previous studies have also concluded that the current status of genetic diversity and structure of natural populations is strongly influenced by suitable niche space fragmentation [26,44]. Therefore, the cultivation of *B. luminifera* is one of the most effective methods for protecting the remaining *B. luminifera* natural populations [32]. In fact, the scholarly probe into climate change has perennially been a subject of contention, yet it is indisputable that any shift in the global climate will have profound ramifications for the existing ecosystem. In this study, employing *B. luminifera* as a representative case, we forecasted the potential impacts of climate change and offered bespoke strategies for an optimal response; this evaluation is as vital as scientific experience, knowledge, and existence itself.

In this study, a macroscopically drawn map of the suitable niche space for *B. luminifera* was developed by combining multi-climate model results (Figure 3 and Figure 4). Our results revealed the intersection region of the suitable habitats at different scenarios (e.g., suitable area in the 2050s, in Figure 6), which should have priority for protection to guarantee habitat survival. For example, we found that the most suitable niche space for *B. luminifera* would severely decrease and move to higher latitudes with climate change. Furthermore, the available ex situ steps for the excellently distributed individuals and seedlings in marginally suitable habitats may be essential to projects by using field survey and provenance testing. The populations at distribution margins should also be given more consideration for management, because they demonstrate potential for the development of climate suitability in the future. Additionally, our research provides a helpful basis for forest management objectives (i.e., reforestation, seed allocation, and assisted migration), which could incorporate climate change adaptation into forest plan programs of *B. luminifera*.

## 4. Materials and Methods

### 4.1. Species Occurrence Database

*B. luminifera* existence information has been collected from published scientific literature and the Chinese Virtual Herbarium (CVH, https://www.cvh.ac.cn/, (accessed on 23 May 2024)). To guarantee the reliability and validity of the occurrence data, only existence points with clear longitude and latitude were selected, and priority was given to records from field surveys.

Based on GIS modeling technology, appropriation geographic point information was further chosen through the following three standards: (1) duplicate coordinate information was removed; considering the resolution of environmental factors, the data were further condensed to ensure that each grid range (evaluation unit) had a single sample site; (2) sampling sites originated from different ecological niches to guarantee balance of species occurrence investigations; and (3) based on meeting the above two principles, some sampling sites were deleted to guarantee that the space between two occurrence data was >10 km as possible and the points were evenly distributed. These measures allowed reducing sample points spatial autocorrelation and minimizing its effect on niche model prediction results.

### 4.2. Environmental Factors Database

Three types of environmental variables (climatical, soil, and ultraviolet B) were collected to simulate *B. luminifera* potential habitat distribution. The climate factors were 19 bioclimatic variables [45], a common dataset usually applied in ecological niche modeling, and originated from the monthly temperature and precipitation values to produce more meteorologically significant climate variables. The soil type database was obtained from the Harmonized World Soil Database (HWSD) [46], and the soil properties of topsoil and subsoil were selected to build the niche model. Ultraviolet radiation B (UV-B) was derived from the gIUV database (http://www.ufz.de/gluv/, (accessed on 23 May 2024)), with six biologically meaningful factors selected to build the niche model.

Future climate scenarios databases were coming from the WorldClim Coupled Model Intercomparison Project 6 (CMIP6) dataset (version 2.0) [47]. In this study, the spatial resolutions of all the baseline and future environment variables were resampled at 30″ (approximately 1 km^2^). Then, we simulated *B. luminifera’s* potential habitat distribution in two future periods: 2041–2060 (2050s) and 2061–2080 (2070s), under the four projected climate scenarios mentioned above [48,49,50]. Each of the two future environmental datasets consisted of four shared socioeconomic pathways (SSPs): SSP126 (slight climate change conditions), SSP245 (moderate climate change conditions), SSP370 (powerful climate change conditions), and SSP585 (severe climate change conditions) [51].

The multicollinearity of environmental variables is expected to cause additional uncertainty for the ecological niche models [11,52]. Indeed, bioclimatic variables have been proven to cause severe multicollinearity [5,6,53]. Hence, a principal component analysis (PCA) was implemented to choose the representative climate factors [4,11]. According to *B. luminifera*, existence point distribution and the forecasting of the 19 bioclimatic factors in the habitat space were delimited by the PCA (Figure S1).

Finally, eight climate variables were chosen to build the climate suitability model (Table S2). Additionally, Pearson product moment correlation coefficients (*r*) were applied to check the cross-correlation of all variables, to ensure that their correlation coefficients were less than 0.6. Ultimately, 8 climatic factors, 12 soil types, and 3 UV-B variables were selected based on PCA and correlation analyses for niche model construction (Table S2).

### 4.3. Construction of Niche-Based GIS Modeling

In this study, a CHS niche-based GIS model was constructed to analyze *B. luminifera* potential habitat distribution under the different climate scenarios (above). This model approach included activities of two distinct patterns: (1) a climate suitability model, which specialized in detecting the dynamic changes of species habitat distribution under future climate scenarios based on EM strategy; and (2) a distribution restricted model, which used the MaxEnt algorithm to predict spread restriction [54]. Although *B. luminifera* has a wide distribution area, the species does not grow in unsuitable soil and ultraviolet environments, even though the climate and landform are suitable. Therefore, in this study, soil and ultraviolet variables were considered limiting ecological factors.

#### 4.3.1. Climate Suitability Model

According to the EM strategy, 10 commonly niche-based GIS modeling algorithms, including the surface range envelope (SRE), flexible discriminant analysis (FDA), generalized linear model (GLM), generalized additive model (GAM), multiple adaptive regression splines (MARS), generalized boosting model (GBM), classification tree analysis (CTA), artificial neural network (ANN), random forest (RF), and maximum entropy (MaxEnt) [54,55,56,57], were utilized to develop the climate suitability model. This approach was adopted to reduce the modeling prediction uncertainty caused by different modeling techniques. All model building steps were implemented on the biomod2 framework based on R language [58]. The modeling process occurred as follows (Figure S2):

Firstly, pseudo-sampling points were selected. For this process, to decrease the prediction result’s instability caused by randomly generated points, this step was reduplicated three times to produce three datasets of pseudo-sampling points, with each dataset containing 500 pseudo-sampling points.

Secondly, we conducted single-model development. Ten simulation algorithms were developed independently using the method of biomod2 in the R language environment. A total of 80% of the sampling points (containing occurrence points and pseudo databases) were freely chosen as training databases, and the remaining 20% was chosen for model testing (i.e., cross-validation). To decrease the instability due to various data, the true skill statistic (TSS) and receiver operating characteristic curve (ROC) were applied to estimate the model representation [59,60], and an individual estimated algorithm was performed 10 times with each sample dataset. Hence, in this process, 120 single models (i.e., 4 single modeling algorithms, 3 pseudo-absence sampling models, and 10 cross-validation runs) were built.

Thirdly, we built the EMs. In this step, mean weight values were adopted to blend all individual models along with TSS values > 0.7 to generate our EMs. Thus, only models with a TSS value > 0.7 were retained to develop the final ensemble. Then, TSS values were used to estimate the references and define the weight values of individual models. Equation (1) presents how the TSS values were utilized to determine the weight of an individual model:

(1) $$W_{j}=\frac{r_{j}}{\sum_{j=1}^{h}r_{j}}$$

$W_{j}$ is the weight value of *j*’s results; $r_{j}$ is the TSS value of model *j*; and *h* is the quantity of models. 

The normalization prediction results of an individual model were then redoubled by the commensurable weight to receive the comprehensive results. Equation (2) illustrates how the potential habitat suitability index was calculated and the EM was constructed.

(2) $${EM}_{i}=\sum_{j=1}^{n}W_{j}\times X_{ij}$$

${EM}_{i}$ (range [0, 1]) means the habitat suitability index of the estimation unit (grid) *i*, which is the estimation index for potential *B. luminifera* habitats; $W_{j}$ is the weight value of *j*’s result; and $X_{ij}$ refers to the value of the estimation grid *i* of model *j*’s result.

Based on the above ensemble model, *B. luminifera’s* potential habitat distribution in the 2000s, 2050s, and 2070s was performed, and among them, the future climate database included four SSPs (SSP126, SSP245, SSP370, and SSP585) scenarios. Based on the future bioclimatic environmental database, an individual model was conducted independently for every period, and the areas for suitable habitat changes in *B. luminifera* under different scenarios/periods were counted. Hence, *B. luminifera’s* future habitat distribution in different stages was achieved by averaging the results.

Finally, the binary format that predicted the results of EMs was obtained. In this process, optimized TSS values based on the testing data were utilized as a reference (threshold) to convert the ensemble modeling’s results to a binary format [11]. Because TSS refers to a threshold-dependent metric, but different thresholds can bring about various TSS values, in this study, the threshold parallel to the maximal TSS results was selected for the taxonomy criteria. Based on a unified threshold, the model’s simulation results in the 2000s, 2050s, and 2070s were divided into two types: suitable and unsuitable.

#### 4.3.2. Distribution Limitation Model

Soil variables and UV-B radiation data can supply messages concerning key environmental variables that constrain species habitats spatially. Among the ecological niche model technologies, only the maximum entropy algorithm can utilize categorical data (e.g., soil texture classification) as input environmental variables. Hence, in order to assess soil and UV-B suitability requirements, we employed 12 soil and 3 UV-B variables to build a habitat distribution limitation model by MaxEnt (version3.3.3) [19,60].

In the modeling steps, 75% existence points from the database were freely selected as training, and the reserving database was applied as testing data. Moreover, 10-time repeats were conducted to minimize the instability, and the AUC (area under the curve) was utilized to assess the model operation representation. The MaxEnt prediction model for the soil variables suitability index scope in 0 to 1 and the maximum training sensitivity plus specificity were considered as the thresholds to reclassify the results into two types: suitable and unsuitable soil habitats. Similarly, MaxEnt was also applied to develop a species habitat spread restriction model to assess its UV-B radiation suitability requirements.

### 4.4. Comprehensive Habitat Suitability (CHS) Model

Here, a CHS model was developed to estimate *B. luminifera’s* suitable habitat distribution, including bioclimate suitability, soil suitability, and UV-B suitability, and this model provided the CHS value for every estimation grid as below (Equation (3)):

(3) $${CHS}_{i}={TM}_{i}⋂S_{i}⋂U_{i}$$

${CHS}_{i}$ refers to the comprehensive habitat suitability conditions of *B. luminifera* of each estimation grid *i*; ${TM}_{i}$ refers to the climate suitability of each estimation grid i under various environment scenarios; $S_{i}$ refers to the soil limitation index of each estimation grid *i*; and $U_{i}$ refers to the UV-B limitation index of each estimation grid *i*. In the modeling operation, the soil and UV-B conditions of the future were considered to maintain consistency with the current period. 

### 4.5. B. luminifera Future Potential Habitats Distribution Areas

The model prediction results contained uncertainties under various future climate scenarios that were generated by various atmospheric general circulation models (GCMs). To reduce the effect of various GCMs, the certainty index of *B. luminifera’s* suitability habitat ($C_{i}$, Equation (4)) was formulated to present the confidence of future prediction as follows:

(4) $$C_{i}=\frac{\sum_{1}^{m}{CHS}_{i}}{m}$$

$C_{i}$ is the certainty index of *B. luminifera’s* suitability habitat in each climate change scenario. In this study, eight climate change scenarios were included; ${CHS}_{i}$ is the comprehensive habitat suitability’s result into estimation grid *i* under the *j*th GCM. *m* is the quantity of GCMs contained in the statistics under this climate change scenario. For each environmental scenario, the regions along with $C_{i}$ values > 0.5 were considered as the final suitable habitats.

## 5. Conclusions

In the present study, a comprehensive habitat suitability (CHS) niche-based GIS model was developed using the ensemble model strategy and applied for predicting the suitable climate habitat geographic range of *B. luminifera*. We found that climate change will negatively affect the spatial distribution of *B. luminifera*, resulting in a northward range shift and a drastic loss of suitable habitats in the future. Our research provides a macroscopically drawn map of potentially suitable areas (plantations) of *B. luminifera* that were in the intersection region at different scenarios, which was more reliable than the individual union–intersection habitat under a single scenario/year. Therefore, the present study provides a valuable basis for reforestation, seed allocation, and assisted migration of *B. luminifera*, and it will ultimately help boost the survival rate of afforestation for *B. luminifera*.

## Figures and Tables

**Table 1:** *B. luminifera* dynamics of habitat area under future climate scenario/year.

Future	Suitable Habitats (×10^6^ km^2^)		
	Unchanged	Gain	Loss
2050s	1.33	0.15	0.18
2070s	1.26	0.10	0.25

**Table 2:** Statistical area for suitable habitats distribution changes for *B*. *luminifera* under different representative concentration pathways (SSPs: SSP1-2.6, SSP2-4.5, SSP3-7.0, and SSP5-8.5) at different times in China.

	2050s (×10^6^ km)			2070s (×10^6^ km)		
	Unchanged	Gain	Loss	Unchanged	Gain	Loss
SSP1-2.6	1.09	0.02	0.42	1.22	0.04	0.30
SSP2-4.5	1.31	0.12	0.20	1.07	0.07	0.44
SSP3-7.0	1.14	0.03	0.38	0.86	0.03	0.65
SSP5-8.5	1.08	0.07	0.43	0.81	0.05	0.71

**Table 3:** The codes for the eight habitat-suitability types.

Code	Climate Condition	Soil Condition	UV-B Condition
I	Unsuitable	Unsuitable	Unsuitable
II	Suitable	Suitable	Suitable
III	Suitable	Suitable	Unsuitable
IV	Suitable	Unsuitable	Suitable
V	Unsuitable	Suitable	Suitable
VI	Suitable	Unsuitable	Unsuitable
VII	Unsuitable	Suitable	Unsuitable
VIII	Unsuitable	Unsuitable	Suitable

**Table 4:** Habitat area with different suitability types of *B. luminifera* at various periods in China. I: unsuitable; II: suitable habitat; III: habitats with an unsuitable UV-B condition; IV: habitats with an unsuitable soil condition; V: habitats with unsuitable climate conditions; VI: habitats with an unsuitable soil and UV-B condition; VII: habitats with an unsuitable climate and UV-B condition; VIII: habitats with unsuitable climate and soil conditions.

Type	Habitats under Future Climate Scenario/Year (×10^6^ km^2^)		
	Current	2050s	2070s
II	1.15	1.07	0.99
III	0.08	0.06	0.06
IV	0.25	0.29	0.25
V	0.24	0.31	0.40
VI	0.05	0.06	0.06
VII	0.50	0.51	0.52
VIII	0.27	0.22	0.25

**Table 5:** Statistical area for climatic habitat suitability changes for *B*. *luminifera* under different representative concentration pathways (SSPs: SSP1-2.6, SSP2-4.5, SSP3-7.0, and SSP5-8.5) at different times in China.

Code	Current	2050s (×10^6^ km^2^)				2070s (×10^6^ km^2^)			
		SSP 1-2.6	SSP 2-4.5	SSP 3-7.0	SSP 5-8.5	SSP 1-2.6	SSP 2-4.5	SSP 3-7.0	SSP 5-8.5
I	7.07	7.09	7.08	7.08	7.08	7.08	7.08	7.11	7.10
II	1.15	0.86	1.05	0.88	0.84	0.95	0.83	0.69	0.63
III	0.08	0.05	0.05	0.05	0.05	0.05	0.05	0.02	0.03
IV	0.25	0.17	0.28	0.19	0.21	0.20	0.22	0.16	0.16
V	0.24	0.53	0.34	0.51	0.55	0.44	0.56	0.70	0.76
VI	0.05	0.04	0.05	0.05	0.05	0.05	0.05	0.02	0.04
VII	0.50	0.52	0.52	0.53	0.53	0.52	0.53	0.55	0.55
VIII	0.27	0.34	0.23	0.31	0.30	0.31	0.29	0.35	0.34

## Data Availability

The original data presented in the study are openly available in Table S1 and Figure 1.

## Supplementary Materials

The following supporting information can be downloaded at https://www.mdpi.com/article/10.3390/plants13111542/s1, Figure S1: The principal component analysis (PCA) process to select a subset of the environmental factors. A, the distribution of the points of *B. luminifera* occurrences in the environmental space defined by the first two PCA axes, illustration of the distribution of *B. luminifera* along the first two PCA axes; B, the correlation circle of the selected bioclimatic variables as a function of these first two PCA axes, illustration of the projection of the selected bioclimatic variables over the same two PCA axes. Figure S2: Schematic representation of the methodological methods. Table S1: The occurrence data of *Betula luminifera*. Table S2: Environmental factors applied to niche-based GIS modeling of *Betula luminifera*. Table S3: Habitat area with different suitability type of *Betula luminifera* at various periods in China. I: Unsuitable; II: Suitable habitat; III: Habitats with unsuitable UV-B condition; IV: Habitats with unsuitable Soil condition; V: Habitats with unsuitable climate conditions; VI: Habitats with unsuitable Soil and UV-B condition; VII: Habitats with unsuitable Climate and UV-B condition; VIII: Habitats with unsuitable Climate and Soil condition. Table S4: Mean and standard deviation of the eight bioclimatic variables in mainland China.
