# Supplementary material for: The Spatial Shifts and Vulnerability Assessment of Ecological Niches under Climate Change Scenarios for Betula luminifera, a Fast-Growing Precious Tree in China
Source: Plants (Basel). 2024 Jun 2;13(11):1542. doi: 10.3390/plants13111542 (PMC11174992; doi:10.3390/plants13111542)
Supplement: Supplementary file 1 [file plants-13-01542-s001.zip › Table S1.pdf]

Table S1. The occurrence data of *Betula luminifera*.

No.	Species	Long/°E	Lat/°N	Source
1	*Betula luminifera*	116.580733	30.669257	Field surveys
2	*Betula luminifera*	118.14761	30.278651	Chinese Virtual Herbarium (https://www.cvh.ac.cn/)
3	*Betula luminifera*	117.550746	29.627456	Field surveys
4	*Betula luminifera*	118.20543	29.795459	Chinese Virtual Herbarium (https://www.cvh.ac.cn/)
5	*Betula luminifera*	116.22439	31.093076	Chinese Virtual Herbarium (https://www.cvh.ac.cn/)
6	*Betula luminifera*	118.585444	30.073292	Chinese Virtual Herbarium (https://www.cvh.ac.cn/)
7	*Betula luminifera*	116.878027	25.652507	Field surveys
8	*Betula luminifera*	116.145217	25.142201	Field surveys
9	*Betula luminifera*	117.437515	25.300759	Field surveys
10	*Betula luminifera*	117.996278	27.746818	Chinese Virtual Herbarium (https://www.cvh.ac.cn/)
11	*Betula luminifera*	117.653289	27.144903	Field surveys
12	*Betula luminifera*	117.594562	26.959805	Chinese Virtual Herbarium (https://www.cvh.ac.cn/)
13	*Betula luminifera*	117.870707	26.97112	Field surveys
14	*Betula luminifera*	118.000941	25.007442	Field surveys
15	*Betula luminifera*	117.577888	25.786708	Field surveys
16	*Betula luminifera*	117.073144	26.440383	Field surveys
17	*Betula luminifera*	117.093439	27.011764	Chinese Virtual Herbarium (https://www.cvh.ac.cn/)
18	*Betula luminifera*	117.136651	25.739144	Field surveys
19	*Betula luminifera*	118.133432	26.037734	Field surveys
20	*Betula luminifera*	117.866643	27.876114	Chinese Virtual Herbarium (https://www.cvh.ac.cn/)
21	*Betula luminifera*	105.43005	32.735093	Chinese Virtual Herbarium (https://www.cvh.ac.cn/)
22	*Betula luminifera*	105.87720	32.980362	Chinese Virtual Herbarium

				(https://www.cvh.ac.cn/)
23	*Betula luminifera*	113.095309	24.926423	Chinese Virtual Herbarium (https://www.cvh.ac.cn/)
24	*Betula luminifera*	114.266726	24.728258	Field surveys
25	*Betula luminifera*	111.948091	23.463971	Chinese Virtual Herbarium (https://www.cvh.ac.cn/)
26	*Betula luminifera*	106.576205	24.353029	Chinese Virtual Herbarium (https://www.cvh.ac.cn/)
27	*Betula luminifera*	105.769382	23.199318	Chinese Virtual Herbarium (https://www.cvh.ac.cn/)
28	*Betula luminifera*	106.290611	24.506727	Chinese Virtual Herbarium (https://www.cvh.ac.cn/)
29	*Betula luminifera*	105.359981	24.158233	Chinese Virtual Herbarium (https://www.cvh.ac.cn/)
30	*Betula luminifera*	106.340276	23.802653	Chinese Virtual Herbarium (https://www.cvh.ac.cn/)
31	*Betula luminifera*	109.951732	25.56688	Chinese Virtual Herbarium (https://www.cvh.ac.cn/)
32	*Betula luminifera*	110.147937	25.883546	Chinese Virtual Herbarium (https://www.cvh.ac.cn/)
33	*Betula luminifera*	110.076497	25.735092	Chinese Virtual Herbarium (https://www.cvh.ac.cn/)
34	*Betula luminifera*	110.070177	26.064963	Chinese Virtual Herbarium (https://www.cvh.ac.cn/)
35	*Betula luminifera*	110.986035	26.075144	Chinese Virtual Herbarium (https://www.cvh.ac.cn/)
36	*Betula luminifera*	110.48338	25.774805	Chinese Virtual Herbarium (https://www.cvh.ac.cn/)
37	*Betula luminifera*	110.720705	25.449309	Chinese Virtual Herbarium (https://www.cvh.ac.cn/)
38	*Betula luminifera*	106.979712	24.552119	Chinese Virtual Herbarium (https://www.cvh.ac.cn/)
39	*Betula luminifera*	108.671316	25.110615	Chinese Virtual Herbarium (https://www.cvh.ac.cn/)
40	*Betula luminifera*	108.574832	25.367083	Chinese Virtual Herbarium (https://www.cvh.ac.cn/)
41	*Betula luminifera*	107.169948	25.0005115	Chinese Virtual Herbarium (https://www.cvh.ac.cn/)
42	*Betula luminifera*	109.707247	25.603041	Chinese Virtual Herbarium (https://www.cvh.ac.cn/)
43	*Betula luminifera*	109.256334	25.065934	Field surveys

44	*Betula luminifera*	110.15489	22.601659	Field surveys
45	*Betula luminifera*	105.736923	26.188698	Chinese Virtual Herbarium (https://www.cvh.ac.cn/)
46	*Betula luminifera*	105.972406	26.251498	Chinese Virtual Herbarium (https://www.cvh.ac.cn/)
47	*Betula luminifera*	105.763471	27.555005	Chinese Virtual Herbarium (https://www.cvh.ac.cn/)
48	*Betula luminifera*	105.871673	27.227254	Chinese Virtual Herbarium (https://www.cvh.ac.cn/)
49	*Betula luminifera*	105.969816	26.628438	Chinese Virtual Herbarium (https://www.cvh.ac.cn/)
50	*Betula luminifera*	106.699008	26.604073	Chinese Virtual Herbarium (https://www.cvh.ac.cn/)
51	*Betula luminifera*	106.753963	26.560957	Chinese Virtual Herbarium (https://www.cvh.ac.cn/)
52	*Betula luminifera*	106.706862	26.900809	Chinese Virtual Herbarium (https://www.cvh.ac.cn/)
53	*Betula luminifera*	106.577613	26.837673	Chinese Virtual Herbarium (https://www.cvh.ac.cn/)
54	*Betula luminifera*	105.291176	26.373946	Chinese Virtual Herbarium (https://www.cvh.ac.cn/)
55	*Betula luminifera*	109.207438	26.682632	Chinese Virtual Herbarium (https://www.cvh.ac.cn/)
56	*Betula luminifera*	109.250244	26.659098	Chinese Virtual Herbarium (https://www.cvh.ac.cn/)
57	*Betula luminifera*	108.089183	26.381846	Chinese Virtual Herbarium (https://www.cvh.ac.cn/)
58	*Betula luminifera*	107.423296	26.398408	Chinese Virtual Herbarium (https://www.cvh.ac.cn/)
59	*Betula luminifera*	108.014977	27.146642	Chinese Virtual Herbarium (https://www.cvh.ac.cn/)
60	*Betula luminifera*	108.306581	26.61791	Chinese Virtual Herbarium (https://www.cvh.ac.cn/)
61	*Betula luminifera*	107.696223	26.053392	Chinese Virtual Herbarium (https://www.cvh.ac.cn/)
62	*Betula luminifera*	106.998216	26.616052	Chinese Virtual Herbarium (https://www.cvh.ac.cn/)
63	*Betula luminifera*	107.965193	25.956172	Chinese Virtual Herbarium (https://www.cvh.ac.cn/)
64	*Betula luminifera*	107.646862	26.985969	Chinese Virtual Herbarium (https://www.cvh.ac.cn/)
65	*Betula luminifera*	105.907439	25.045371	Chinese Virtual Herbarium (https://www.cvh.ac.cn/)

66	*Betula luminifera*	106.106445	25.184355	Chinese Virtual Herbarium (https://www.cvh.ac.cn/)
67	*Betula luminifera*	105.13627	25.198038	Chinese Virtual Herbarium (https://www.cvh.ac.cn/)
68	*Betula luminifera*	105.845975	25.183212	Chinese Virtual Herbarium (https://www.cvh.ac.cn/)
69	*Betula luminifera*	108.110057	28.567949	Chinese Virtual Herbarium (https://www.cvh.ac.cn/)
70	*Betula luminifera*	108.39615	28.529511	Chinese Virtual Herbarium (https://www.cvh.ac.cn/)
71	*Betula luminifera*	108.157415	27.354372	Chinese Virtual Herbarium (https://www.cvh.ac.cn/)
72	*Betula luminifera*	108.544061	27.520892	Chinese Virtual Herbarium (https://www.cvh.ac.cn/)
73	*Betula luminifera*	108.860245	28.04065	Chinese Virtual Herbarium (https://www.cvh.ac.cn/)
74	*Betula luminifera*	109.14885	27.787174	Chinese Virtual Herbarium (https://www.cvh.ac.cn/)
75	*Betula luminifera*	105.754068	28.361585	Chinese Virtual Herbarium (https://www.cvh.ac.cn/)
76	*Betula luminifera*	107.619484	28.86865	Chinese Virtual Herbarium (https://www.cvh.ac.cn/)
77	*Betula luminifera*	106.527831	28.546565	Chinese Virtual Herbarium (https://www.cvh.ac.cn/)
78	*Betula luminifera*	106.487153	27.873588	Chinese Virtual Herbarium (https://www.cvh.ac.cn/)
79	*Betula luminifera*	107.192477	27.964304	Chinese Virtual Herbarium (https://www.cvh.ac.cn/)
80	*Betula luminifera*	106.522067	28.229194	Chinese Virtual Herbarium (https://www.cvh.ac.cn/)
81	*Betula luminifera*	107.328606	28.3214	Chinese Virtual Herbarium (https://www.cvh.ac.cn/)
82	*Betula luminifera*	106.61001	27.626018	Chinese Virtual Herbarium (https://www.cvh.ac.cn/)
83	*Betula luminifera*	111.866841	33.6149042	Chinese Virtual Herbarium (https://www.cvh.ac.cn/)
84	*Betula luminifera*	111.855425	33.051203	Chinese Virtual Herbarium (https://www.cvh.ac.cn/)
85	*Betula luminifera*	110.212668	30.091449	Chinese Virtual Herbarium (https://www.cvh.ac.cn/)
86	*Betula luminifera*	109.015795	30.138724	Chinese Virtual Herbarium (https://www.cvh.ac.cn/)
87	*Betula luminifera*	109.758063	30.030343	Chinese Virtual Herbarium (https://www.cvh.ac.cn/)

88	*Betula luminifera*	116.040984	30.978153<br>5	Chinese Virtual Herbarium (https://www.cvh.ac.cn/)
89	*Betula luminifera*	110.682525	31.750496	Chinese Virtual Herbarium (https://www.cvh.ac.cn/)
90	*Betula luminifera*	111.240689	32.494669	Chinese Virtual Herbarium (https://www.cvh.ac.cn/)
91	*Betula luminifera*	110.964396	31.014361	Chinese Virtual Herbarium (https://www.cvh.ac.cn/)
92	*Betula luminifera*	110.960682	31.047633	Chinese Virtual Herbarium (https://www.cvh.ac.cn/)
93	*Betula luminifera*	110.357081	30.192189	Chinese Virtual Herbarium (https://www.cvh.ac.cn/)
94	*Betula luminifera*	110.45063	30.39108	Chinese Virtual Herbarium (https://www.cvh.ac.cn/)
95	*Betula luminifera*	110.914934	30.045428	Chinese Virtual Herbarium (https://www.cvh.ac.cn/)
96	*Betula luminifera*	111.185777	28.557195	Chinese Virtual Herbarium (https://www.cvh.ac.cn/)
97	*Betula luminifera*	112.932379	24.988985	Chinese Virtual Herbarium (https://www.cvh.ac.cn/)
98	*Betula luminifera*	112.875563	27.235996	Chinese Virtual Herbarium (https://www.cvh.ac.cn/)
99	*Betula luminifera*	112.735781	27.258767	Chinese Virtual Herbarium (https://www.cvh.ac.cn/)
100	*Betula luminifera*	110.163102	27.195552<br>5	Chinese Virtual Herbarium (https://www.cvh.ac.cn/)
101	*Betula luminifera*	110.801765	27.57312	Chinese Virtual Herbarium (https://www.cvh.ac.cn/)
102	*Betula luminifera*	110.377973	28.191351	Chinese Virtual Herbarium (https://www.cvh.ac.cn/)
103	*Betula luminifera*	110.459161	28.787976	Chinese Virtual Herbarium (https://www.cvh.ac.cn/)
104	*Betula luminifera*	110.78693	28.533734	Chinese Virtual Herbarium (https://www.cvh.ac.cn/)
105	*Betula luminifera*	110.56114	28.234584	Chinese Virtual Herbarium (https://www.cvh.ac.cn/)
106	*Betula luminifera*	109.691402	27.449513	Chinese Virtual Herbarium (https://www.cvh.ac.cn/)
107	*Betula luminifera*	110.328537	26.396203	Chinese Virtual Herbarium (https://www.cvh.ac.cn/)
108	*Betula luminifera*	110.31408	26.364458<br>1	Chinese Virtual Herbarium (https://www.cvh.ac.cn/)
109	*Betula luminifera*	110.582579	27.065975	Chinese Virtual Herbarium (https://www.cvh.ac.cn/)

110	*Betula luminifera*	110.100227	26.413673	Chinese Virtual Herbarium (https://www.cvh.ac.cn/)
111	*Betula luminifera*	110.620506	26.671774	Chinese Virtual Herbarium (https://www.cvh.ac.cn/)
112	*Betula luminifera*	110.936523	26.421437	Chinese Virtual Herbarium (https://www.cvh.ac.cn/)
113	*Betula luminifera*	110.639679	26.52059	Chinese Virtual Herbarium (https://www.cvh.ac.cn/)
114	*Betula luminifera*	111.464449	27.327132	Chinese Virtual Herbarium (https://www.cvh.ac.cn/)
115	*Betula luminifera*	109.466399	28.632099	Chinese Virtual Herbarium (https://www.cvh.ac.cn/)
116	*Betula luminifera*	109.745577	28.317369	Chinese Virtual Herbarium (https://www.cvh.ac.cn/)
117	*Betula luminifera*	110.09139	28.663066	Chinese Virtual Herbarium (https://www.cvh.ac.cn/)
118	*Betula luminifera*	109.294181	29.218692	Chinese Virtual Herbarium (https://www.cvh.ac.cn/)
119	*Betula luminifera*	109.83907	29.186572	Chinese Virtual Herbarium (https://www.cvh.ac.cn/)
120	*Betula luminifera*	109.655037	28.967425	Chinese Virtual Herbarium (https://www.cvh.ac.cn/)
121	*Betula luminifera*	116.413384	39.910925	Chinese Virtual Herbarium (https://www.cvh.ac.cn/)
122	*Betula luminifera*	111.764466	28.06206	Field surveys
123	*Betula luminifera*	111.379736	25.484495	Chinese Virtual Herbarium (https://www.cvh.ac.cn/)
124	*Betula luminifera*	111.658977	24.796354	Chinese Virtual Herbarium (https://www.cvh.ac.cn/)
125	*Betula luminifera*	111.722946	25.981665	Chinese Virtual Herbarium (https://www.cvh.ac.cn/)
126	*Betula luminifera*	113.904966	28.620455	Chinese Virtual Herbarium (https://www.cvh.ac.cn/)
127	*Betula luminifera*	110.490013	29.376381	Chinese Virtual Herbarium (https://www.cvh.ac.cn/)
128	*Betula luminifera*	109.816625	29.620995	Chinese Virtual Herbarium (https://www.cvh.ac.cn/)
129	*Betula luminifera*	109.816396	29.685865	Chinese Virtual Herbarium (https://www.cvh.ac.cn/)
130	*Betula luminifera*	110.45065	29.332753	Chinese Virtual Herbarium (https://www.cvh.ac.cn/)
131	*Betula luminifera*	110.477219	29.359047	Chinese Virtual Herbarium (https://www.cvh.ac.cn/)

132	*Betula luminifera*	110.441345	29.321096	Chinese Virtual Herbarium (https://www.cvh.ac.cn/)
133	*Betula luminifera*	114.092418	25.394517	Field surveys
134	*Betula luminifera*	114.577536	24.677223	Field surveys
135	*Betula luminifera*	117.23484	29.551782	Field surveys
136	*Betula luminifera*	114.069302	29.032227	Chinese Virtual Herbarium (https://www.cvh.ac.cn/)
137	*Betula luminifera*	114.739691	28.792048	Chinese Virtual Herbarium (https://www.cvh.ac.cn/)
138	*Betula luminifera*	114.04232	27.358486	Chinese Virtual Herbarium (https://www.cvh.ac.cn/)
139	*Betula luminifera*	114.20558	27.584389	Chinese Virtual Herbarium (https://www.cvh.ac.cn/)
140	*Betula luminifera*	117.847834	29.262921	Field surveys
141	*Betula luminifera*	117.980497	28.893284	Field surveys
142	*Betula luminifera*	114.724886	27.775471	Chinese Virtual Herbarium (https://www.cvh.ac.cn/)
143	*Betula luminifera*	115.104982	28.831342	Chinese Virtual Herbarium (https://www.cvh.ac.cn/)
144	*Betula luminifera*	114.299989	27.609983	Chinese Virtual Herbarium (https://www.cvh.ac.cn/)
145	*Betula luminifera*	114.605692	28.53966	Chinese Virtual Herbarium (https://www.cvh.ac.cn/)
146	*Betula luminifera*	117.214109	28.293483	Chinese Virtual Herbarium (https://www.cvh.ac.cn/)
147	*Betula luminifera*	117.363972	27.915077	Chinese Virtual Herbarium (https://www.cvh.ac.cn/)
148	*Betula luminifera*	109.071558	32.287879	Chinese Virtual Herbarium (https://www.cvh.ac.cn/)
149	*Betula luminifera*	108.86807	32.105031	Chinese Virtual Herbarium (https://www.cvh.ac.cn/)
150	*Betula luminifera*	109.315839	32.019855	Chinese Virtual Herbarium (https://www.cvh.ac.cn/)
151	*Betula luminifera*	109.368547	32.394804	Chinese Virtual Herbarium (https://www.cvh.ac.cn/)
152	*Betula luminifera*	108.954598	32.453623	Chinese Virtual Herbarium (https://www.cvh.ac.cn/)
153	*Betula luminifera*	108.616974	34.124907	Chinese Virtual Herbarium (https://www.cvh.ac.cn/)

154	*Betula luminifera*	109.533497	31.889643	Chinese Virtual Herbarium (https://www.cvh.ac.cn/)
155	*Betula luminifera*	107.821651	34.061745	Chinese Virtual Herbarium (https://www.cvh.ac.cn/)
156	*Betula luminifera*	107.02943	33.0738	Chinese Virtual Herbarium (https://www.cvh.ac.cn/)
157	*Betula luminifera*	107.398402	31.986784	Chinese Virtual Herbarium (https://www.cvh.ac.cn/)
158	*Betula luminifera*	103.288345	30.795023	Chinese Virtual Herbarium (https://www.cvh.ac.cn/)
159	*Betula luminifera*	103.554995	30.961146	Chinese Virtual Herbarium (https://www.cvh.ac.cn/)
160	*Betula luminifera*	103.637532	31.096114	Chinese Virtual Herbarium (https://www.cvh.ac.cn/)
161	*Betula luminifera*	102.321234	30.560742	Chinese Virtual Herbarium (https://www.cvh.ac.cn/)
162	*Betula luminifera*	103.356274	29.574322	Chinese Virtual Herbarium (https://www.cvh.ac.cn/)
163	*Betula luminifera*	103.803082	28.423442	Chinese Virtual Herbarium (https://www.cvh.ac.cn/)
164	*Betula luminifera*	105.851986	28.00712	Chinese Virtual Herbarium (https://www.cvh.ac.cn/)
165	*Betula luminifera*	105.819142	28.044758	Chinese Virtual Herbarium (https://www.cvh.ac.cn/)
166	*Betula luminifera*	105.754725	28.185745	Chinese Virtual Herbarium (https://www.cvh.ac.cn/)
167	*Betula luminifera*	105.792596	28.22771	Chinese Virtual Herbarium (https://www.cvh.ac.cn/)
168	*Betula luminifera*	105.451175	28.161786	Chinese Virtual Herbarium (https://www.cvh.ac.cn/)
169	*Betula luminifera*	101.976982	26.285725	Chinese Virtual Herbarium (https://www.cvh.ac.cn/)
170	*Betula luminifera*	103.379602	29.910616	Chinese Virtual Herbarium (https://www.cvh.ac.cn/)
171	*Betula luminifera*	104.511681	32.075049	Chinese Virtual Herbarium (https://www.cvh.ac.cn/)
172	*Betula luminifera*	104.738503	32.472441	Chinese Virtual Herbarium (https://www.cvh.ac.cn/)
173	*Betula luminifera*	104.726656	32.51195	Chinese Virtual Herbarium (https://www.cvh.ac.cn/)
174	*Betula luminifera*	98.7850822	28.4706866	Chinese Virtual Herbarium (https://www.cvh.ac.cn/)
175	*Betula luminifera*	99.037049	27.766766	Chinese Virtual Herbarium (https://www.cvh.ac.cn/)

176	*Betula luminifera*	100.369523	27.623832	Chinese Virtual Herbarium (https://www.cvh.ac.cn/)
177	*Betula luminifera*	104.146179	28.368155	Chinese Virtual Herbarium (https://www.cvh.ac.cn/)
178	*Betula luminifera*	105.486676	23.522035	Chinese Virtual Herbarium (https://www.cvh.ac.cn/)
179	*Betula luminifera*	104.810768	23.184049	Chinese Virtual Herbarium (https://www.cvh.ac.cn/)
180	*Betula luminifera*	104.738107	23.168727	Chinese Virtual Herbarium (https://www.cvh.ac.cn/)
181	*Betula luminifera*	104.23504	28.557815	Chinese Virtual Herbarium (https://www.cvh.ac.cn/)
182	*Betula luminifera*	118.50565	29.573008	Field surveys
183	*Betula luminifera*	119.510677	29.547577	Field surveys
184	*Betula luminifera*	119.731517	30.239806	Chinese Virtual Herbarium (https://www.cvh.ac.cn/)
185	*Betula luminifera*	119.64243	27.979554	Chinese Virtual Herbarium (https://www.cvh.ac.cn/)
186	*Betula luminifera*	119.21612	27.939284	Chinese Virtual Herbarium (https://www.cvh.ac.cn/)
187	*Betula luminifera*	118.901206	28.3723	Chinese Virtual Herbarium (https://www.cvh.ac.cn/)
188	*Betula luminifera*	119.415279	28.727444	Chinese Virtual Herbarium (https://www.cvh.ac.cn/)
189	*Betula luminifera*	121.04204	29.162747	Chinese Virtual Herbarium (https://www.cvh.ac.cn/)
190	*Betula luminifera*	107.223082	29.144903	Chinese Virtual Herbarium (https://www.cvh.ac.cn/)
191	*Betula luminifera*	108.530014	31.88327	Chinese Virtual Herbarium (https://www.cvh.ac.cn/)
192	*Betula luminifera*	109.470473	31.024602	Chinese Virtual Herbarium (https://www.cvh.ac.cn/)
193	*Betula luminifera*	106.383644	29.043244	Chinese Virtual Herbarium (https://www.cvh.ac.cn/)
194	*Betula luminifera*	106.372579	28.638952	Chinese Virtual Herbarium (https://www.cvh.ac.cn/)
195	*Betula luminifera*	107.110844	29.158282	Chinese Virtual Herbarium (https://www.cvh.ac.cn/)
196	*Betula luminifera*	107.215026	29.135236	Chinese Virtual Herbarium (https://www.cvh.ac.cn/)
197	*Betula luminifera*	106.692344	29.145665	Chinese Virtual Herbarium (https://www.cvh.ac.cn/)

198	*Betula luminifera*	106.062436	29.781067	Chinese Virtual Herbarium (https://www.cvh.ac.cn/)
199	*Betula luminifera*	108.758437	30.668486	Chinese Virtual Herbarium (https://www.cvh.ac.cn/)
200	*Betula luminifera*	109.997701	31.402344	Chinese Virtual Herbarium (https://www.cvh.ac.cn/)
201	*Betula luminifera*	107.845723	29.048033	Chinese Virtual Herbarium (https://www.cvh.ac.cn/)
202	*Betula luminifera*	107.685198	29.432724	Chinese Virtual Herbarium (https://www.cvh.ac.cn/)
203	*Betula luminifera*	108.806112	29.48746	Chinese Virtual Herbarium (https://www.cvh.ac.cn/)
204	*Betula luminifera*	108.770494	31.426707	Chinese Virtual Herbarium (https://www.cvh.ac.cn/)
