# Supplementary material for: The Spatial Shifts and Vulnerability Assessment of Ecological Niches under Climate Change Scenarios for Betula luminifera, a Fast-Growing Precious Tree in China
Source: Plants (Basel). 2024 Jun 2;13(11):1542. doi: 10.3390/plants13111542 (PMC11174992; doi:10.3390/plants13111542)
Supplement: Supplementary file 1 [file plants-13-01542-s001.zip › Table S2.pdf]

Table S2. Environmental variables used to predict the potential geographic distribution of *Betula luminifera* habitats.

Classification and source	Code	Description (units)
Climatic variables WorldClim database (http://www.worldclim.org/)	Bio1	Annual mean air temperature (℃)
	Bio2	Mean diurnal temperature range (℃)
	Bio3	Isothermality (%)
	Bio7	Temperature annual range (℃)
	Bio8	Mean temperature of the wettest quarter (℃)
	Bio12	Annual precipitation (mm)
	Bio15	Precipitation seasonality (%)
	Bio18	Precipitation of the warmest quarter (mm)
Soil variables Harmonized World Soil Database(http://www.fao.org/soils-portal)	T_BULK_DENSITY	Topsoil Bulk Density (kg/dm^3)
	T_CEC_CLAY	Topsoil CEC Clay (cmol/kg)
	T_CEC_SOIL	Topsoil CEC Soil (cmol/kg)
	T_CLAY	Topsoil Clay Fraction (%)
	T_ECE	Topsoil Salinity (dS/m)
	T_ESP	Topsoil Sodicity (%)
	T_GRAVEL	Topsoil Gravel Content (%)
	T_OC	Topsoil Organic Carbon (%)
	T_REF_BULK_DENSITY	Topsoil Bulk Density (kg/dm^3)
	T_SILT	Topsoil Silt Fraction (%)
	T_TEXTURE	Topsoil Texture Classification
	T_USDA	Topsoil USDA Texture Classification (%)
Ultraviolet radiation B (UV-B) glUV: A global UV-B radiation dataset for macroecological studies (https://www.ufz.de/gluv)	UV-B2	UV-B Seasonality ($J \cdot m^{-2} \cdot d^{-1}$)
	UV-B3	Mean UV-B of Highest Month ($J \cdot m^{-2} \cdot d^{-1}$)
	UV-B4	Mean UV-B of Lowest Month ($J \cdot m^{-2} \cdot d^{-1}$)
