# Supplementary material for: The Spatial Shifts and Vulnerability Assessment of Ecological Niches under Climate Change Scenarios for Betula luminifera, a Fast-Growing Precious Tree in China
Source: Plants (Basel). 2024 Jun 2;13(11):1542. doi: 10.3390/plants13111542 (PMC11174992; doi:10.3390/plants13111542)
Supplement: Supplementary file 1 [file plants-13-01542-s001.zip › Table S3.pdf]

Table S3. Habitat area with different suitability type of *Betula luminifera* at various periods in China. I: Unsuitable; II: Suitable habitat; III: Habitats with unsuitable UV-B condition; IV: Habitats with unsuitable Soil condition; V: Habitats with unsuitable climate conditions; VI: Habitats with unsuitable Soil and UV-B condition; VII: Habitats with unsuitable Climate and UV-B condition; VIII: Habitats with unsuitable Climate and Soil condition.

Climate change scenario	Average temperature rise (℃)	Suitable habitat area changes rate (%)							
		I	II	III	IV	V	VI	VII	VIII
SSP126-2050s	1.92	3.45	-25.46	-32.41	-32.59	122.22	-10.18	3.75	26.92
SSP245-2050s	2.02	1.15	-8.50	-31.62	13.14	41.31	8.56	3.63	-15.04
SSP370-2050s	2.00	3.13	-23.05	-39.29	-20.81	110.73	5.25	4.80	16.12
SSP585-2050s	2.61	3.60	-26.53	-40.42	-16.18	127.17	13.91	4.97	11.87
SSP126-2070s	1.70	2.28	-16.80	-30.31	-19.02	81.09	20.15	3.43	14.47
SSP245-2070s	2.66	3.80	-27.97	-38.34	-11.70	134.13	18.69	4.65	7.75
SSP370-2070s	3.18	5.44	-40.06	-74.79	-36.66	191.56	-44.93	10.21	30.66
SSP585-2070s	3.62	6.08	-44.81	-63.71	-34.72	214.09	-21.76	8.52	28.88
