# Supplementary material for: The Spatial Shifts and Vulnerability Assessment of Ecological Niches under Climate Change Scenarios for Betula luminifera, a Fast-Growing Precious Tree in China
Source: Plants (Basel). 2024 Jun 2;13(11):1542. doi: 10.3390/plants13111542 (PMC11174992; doi:10.3390/plants13111542)
Supplement: Supplementary file 1 [file plants-13-01542-s001.zip › Table S4.pdf]

Table S4. Mean and standard deviation of the eight bioclimatic variables in mainland China.

		bio1/℃	bio2/℃	bio3/%	bio7/℃	bio8/℃	bio12/mm	bio15/%	bio18/mm
2000S		15.39±2.81	7.92±0.96	28.53±4.44	28.00±2.80	21.81±2.71	1318.29±277.46	65.75±11.94	549.79±106.53
2050s	SSP1-2.6	17.31±2.80	8.54±0.91	29.66±4.32	29.05±2.88	23.52±2.57	1294.68±254.17	66.33±11.35	514.67±98.96
	SSP2-4.5	17.41±2.73	8.24±0.90	29.97±4.14	27.69±2.51	23.76±2.65	1387.48±270.34	68.14±11.76	588.32±119.11
	SSP3-7.0	17.39±2.72	8.39±0.90	29.78±4.64	28.50±2.86	24.45±2.81	1315.00±258.45	67.36±12.59	565.61±106.24
	SSP5-8.5	18.00±2.746	8.28±0.92	29.67±4.10	28.11±2.61	24.90±2.71	1367.99±275.49	70.48±11.53	609.03±123.29
	**Average**	17.53	8.36	29.77	28.33	24.16	1341.29	68.08	569.41
2070s	SSP1-2.6	17.09±2.77	8.21±0.96	28.90±4.28	28.66±2.95	23.47±2.72	1409.08±283.69	68.13±12.15	586.00±127.89
	SSP2-4.5	18.05±2.76	8.50±0.89	30.43±3.77	28.10±2.45	24.48±2.76	1350.08±262.85	67.10±11.77	560.15±103.87
	SSP3-7.0	18.57±2.69	8.73±0.91	30.71±4.30	28.67±2.71	24.95±2.67	1385.74±306.58	71.25±12.65	592.19±132.87
	SSP5-8.5	19.01±2.68	8.32±0.98	29.58±4.30	28.34±2.64	25.76±2.80	1481.92±285.67	66.84±12.33	613.92±133.37
	**Average**	18.18	8.44	29.90	28.44	24.67	1406.70	68.33	588.06
