# Supplementary figures and images for: The Spatial Shifts and Vulnerability Assessment of Ecological Niches under Climate Change Scenarios for Betula luminifera, a Fast-Growing Precious Tree in China
Source: Plants (Basel). 2024 Jun 2;13(11):1542. doi: 10.3390/plants13111542 (PMC11174992; doi:10.3390/plants13111542)

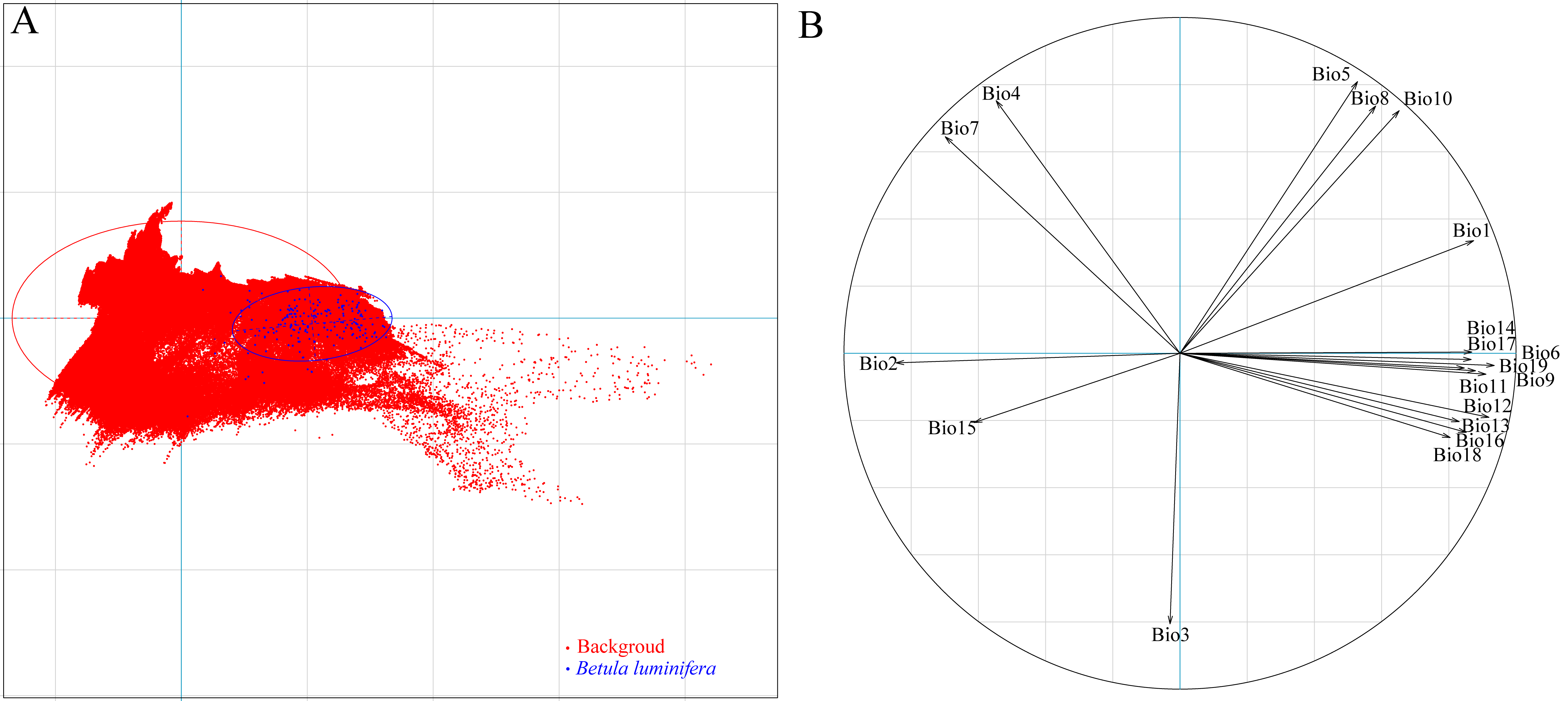

Supplement: Supplementary file 1 [file plants-13-01542-s001.zip › Figure S1.tif]

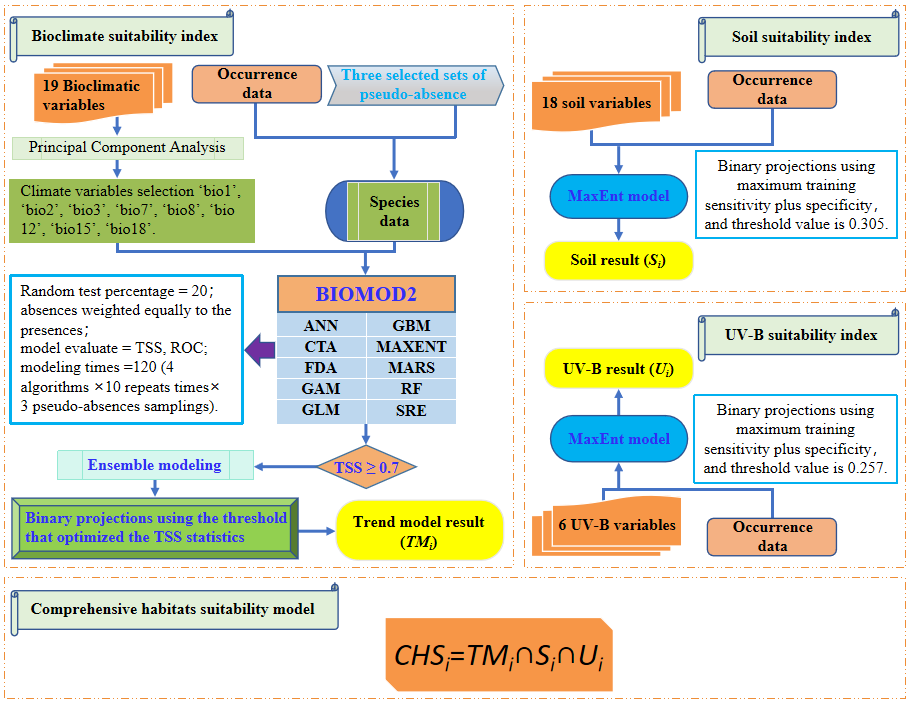

Supplement: Supplementary file 1 [file plants-13-01542-s001.zip › Figure S2.tif]
